# NFAT3-FasL axis synchronously regulates apoptosis and necroptosis in murine cochlear outer hair cells after noise trauma

**DOI:** 10.3389/fnmol.2024.1422646

**Published:** 2024-07-15

**Authors:** Wenlun Wang, Lisheng Yu, Shichang Li, Lin Han, Hongwei Zheng

**Affiliations:** ^1^Department of Otorhinolaryngology-Head and Neck Surgery, Peking University People’s Hospital, Beijing, China; ^2^Department of Otorhinolaryngology, NHC Key Laboratory of Otorhinolaryngology (Shandong University), Qilu Hospital of Shandong University, Jinan, Shandong, China; ^3^Key Laboratory for Experimental Teratology of the Ministry of Education and Department of Pathology, School of Basic Medical Sciences, Cheeloo College of Medicine, Shandong University, Jinan, China; ^4^Department of Otorhinolaryngology-Head and Neck Surgery, Beijing Tsinghua Changgung Hospital, School of Clinical Medicine, Tsinghua University, Beijing, China

**Keywords:** noise-induced hearing loss, apoptosis, necroptosis, NFAT, FasL, FK506, 11R-vivit

## Abstract

Existing studies have indicated that noise induces apoptosis and necroptosis in cochlear outer hair cells (OHCs). However, the role of the extrinsic cell death pathway, initiated by death ligands in the cochlea, remains unknown. In this study, we hypothesized that noise could induce the NFAT3/FasL axis-mediated extrinsic death pathway in the cochlea. We found that NFAT3/FasL signaling was silent in normal OHCs. Noise exposure induced apoptosis and necroptosis in OHCs with specifically high FasL expression. Multiplex immunofluorescence staining revealed that NFAT3 nuclear translocation and FasL upregulation were colocalized in the apoptotic and necroptotic OHCs following noise trauma. Administration of FK506 or 11R-vivit (an specific NFAT inhibitor) blocked NFAT3 nuclear translocation, inhibited FasL expression, mitigated apoptosis and necroptosis, and protected against noise-induced hearing loss (NIHL). Finally, FasL knockdown by delivering siRNA intratympanically attenuated apoptosis and necroptosis in OHCs and alleviated NIHL, confirming the role of FasL in OHC death. Collectively, our study demonstrates that the NFAT3/FasL axis mediates noise-induced extrinsic death pathway in OHCs, leading to their apoptosis and necroptosis.

## 1 Introduction

Noise-induced hearing loss (NIHL) is a significant cause of hearing impairment in adults due to entertainment and occupational acoustic hyperstimulation (Basner et al., 2014). NIHL can accumulate over time and exacerbate the effects of presbycusis (Bowl and Dawson, 2019; Zhuang et al., 2020). The cochlear hair cells, consisting of one row of inner hair cells (IHCs) and three rows of outer hair cells (OHCs), are vital for detecting and translating acoustic vibrations into electrical signals in the auditory system. Due to the non-renewable nature of sensory hair cells, their death and subsequent loss, particularly among the OHCs, constitute the major cause of NIHL (Hayes et al., 2019; Zhang et al., 2020). Research has revealed that the fundamental pathophysiological mechanism underlying NIHL involves the interplay between oxidative stress, Ca2+ overload, and the initiation of death pathways in sensory hair cells (Kurabi et al., 2017; He et al., 2022). It has been suggested that intervening in the regulatory death pathways could alleviate the loss of hair cells and enhance protection against NIHL (Wu et al., 2020; An et al., 2022; Fan et al., 2023). Therefore, acquiring a comprehensive understanding of the mechanisms behind sensory hair cell death has immense potential for developing targeted interventions and medications to prevent noise-induced hearing loss.

The intrinsic apoptosis pathway of sensory hair cells has been extensively studied (Han et al., 2015). However, direct evidence supporting the extrinsic pathway in sensory hair cells is still scarce. Zheng’s et al. (2014) study suggested that the necroptosis of OHCs was implicated in NIHL. The canonical necroptosis pathway is initiated through the engagement of death ligands (Galluzzi et al., 2017). Thus, we hypothesized that the activation of death receptors plays a role in the death of sensory hair cells. FasL, a trimeric transmembrane protein death ligand primarily found on the surface of lymphocytes, is essential for initiating the programmed cell death pathway when it binds to its receptor, FAS. The FasL/Fas-dependent death mechanism enables the immune system to eliminate pathogen-infected cells and tumor cells (Bień et al., 2015). Bodmer et al. (2003) reported that aseptic labyrinthitis could trigger FasL expression in the cochlea. Generally, FasL activates the extrinsic death pathway in neighboring cells possessing Fas receptors. A limited number of cell types, such as T-cell hybridomas and Jurkat cells, undergo autonomous cell death through the FasL–Fas interaction (Brunner et al., 1995).

NFAT3 (Nuclear factor of activated T cells 3), a member of the NFAT transcription factor family, is regulated by Ca^2+^/calcineurin signaling. Calcineurin can induce NFAT dephosphorylation, resulting in the transcription of a series of target genes, including FasL, IL-2 and TNF-α. Intriguingly, the production of reactive oxygen species (ROS) and Ca^2+^ overload, commonly observed in NIHL (Mao and Chen, 2021), has been found to serve as potent stimuli for the dephosphorylation and nucleus translocation of NFAT (Sena et al., 2013). NFAT3 is a common subtype of NFAT found in the nervous system (Mackiewicz et al., 2023). It has been reported that the NFAT3/FasL signaling pathway mediated apoptosis in mouse neurons induced by lithium treatment (Gomez-Sintes and Lucas, 2010). Collectively, these findings suggest that the NFAT3/FasL signaling pathway could be a promising upstream mechanism contributing to the death of sensory hair cells.

Consequently, this study aimed to evaluate the contribution of the NFAT3/FasL axis in noise-induced sensory hair cell death. We utilized pharmacological inhibitors and siRNA silencing to investigate the role of NFAT3 and FasL in noise-induced sensory hair cell death and hearing loss in C57BL/6 J mice. Multiple immunofluorescence staining was combined with cochlear basilar membrane surface preparations to investigate the regulatory cell death mechanism in sensory hair cells. Our results indicated that the NFAT3/FasL signaling is the upstream pathway modulating both the apoptosis and necroptosis of OHCs after noise exposure. During this process, OHCs undergo autonomous cell death through autocrine secretion of the death ligand FasL.

## 2 Materials and methods

### 2.1 Animals

Five-week-old male C57BL/6 J mice were purchased from SPF (Beijing) Biotechnology Co.,Ltd. and acclimatized to their new animal house for 1 week before noise exposure. The temperature in the animal house was maintained at 25 ± 2°C with a 12/12 h day/night cycle. Baseline auditory brainstem responses (ABRs) were measured on the day 3 after arriving at the animal house. All research protocols were approved by the Institutional Animal Care and Use Committee (IACUC) at PKUPH (approval number 2021PHE019).

### 2.2 Noise exposure

Mice at the age of 6 weeks were placed in a stainless steel cage fitted beneath a loudspeaker. The noise was generated by a loudspeaker (Aijie Audio Equipment Factory) driven by a power amplifier (MF-1201 MOSTET, ATech) and an attenuator (PA5 TDT, Alachua, FL, United States). We used white noise at 108 dB sound pressure level (SPL) for 2 h to induce a permanent threshold shift (PTS). The noise intensity at the location of the animal’s head was calibrated using a sound level meter (Model 1,200; Quest Technologies) before and after noise exposure to ensure uniformity. The ambient background noise around the cage was 45 dB. The control mice were kept in the same cage and noise exposure chamber without turning on the noise for 2 h.

### 2.3 Intraperitoneal (IP) drug administration

FK506 (apexbio) was dissolved in DMSO and stored at −20°C as a stock solution (25 mg/mL). 11R-vivit (merck) was dissolved in normal saline and stored at −20°C as a stock solution (1 mg/mL). The two stock solutions were diluted with normal saline before injection. Based on previous studies (Zhang et al., 2013; He et al., 2021), the dose per injection of FK506 and 11R-vivit was 5 mg/kg and 1 mg/kg, respectively. In experiments involving ABR tests and hair cell quantification, mice were treated with intraperitoneal (IP) injections twice daily, starting 2 days before noise exposure and continuing until 2 days after. The mice were then sacrificed 2 weeks after the noise exposure for further analysis. Specifically, on the day of the noise exposure, two IP injections were administered, one hour before and immediately after the exposure. In total, the mice received a total of ten IP injections throughout the experimental period. For immunohistochemistry and western blot analyses, mice were given IP injections twice daily for 2 days prior to the noise exposure. Additionally, injections were administered 1 h before and immediately after the noise exposure, resulting in a total of six IP injections. The mice were then sacrificed at the specified time points. A flow chart illustrating the noise exposure and intraperitoneal administration is depicted in Supplementary Figure S1.

### 2.4 Auditory brainstem responses (ABRs)

Animals were anesthetized with an IP injection of ketamine (100 mg/kg) and xylazine (10 mg/kg) and placed in a sound-isolated and electromagnetically shielded room. A warming pad was used to maintain body temperature. Subdermal electrodes were placed at the vertex of the skull (recording electrode), under the stimulated ear (negative electrode), and the unstimulated ear (ground electrode). Tucker Davis Technology (TDT) System III hardware and SigGen/Biosig software were used to perform ABR tests. ABR thresholds were measured at frequencies of 8, 16 and 32 kHz based on visible and reproducible wave II. Detailed methods have been previously described (Xing et al., 2023). The auditory threshold was measured 4 days before and 14 days after noise exposure, respectively. All ABR tests were performed by one expert who was blind to the experimental conditions.

### 2.5 Immunocytochemistry for cochlear surface preparations and cryosections

Temporal bones were removed immediately after mice were sacrificed by cervical dislocation. Cochleae were perfused with 4% paraformaldehyde in phosphate-buffered saline at 4°C overnight (He et al., 2021). After being rinsed in PBS, they were decalcified with a 10% EDTA decalcifying solution at room temperature (RT) overnight. For surface preparation, decalcified cochleae were dissected under a microscope to remove the otic capsule, spiral ligament, stria vascularis, vestibular membrane and tectorial membrane. Finally, the remaining modiolus and three turns of sensory epithelia were obtained. To prepare cochlear cryosection, decalcified cochleae were dehydrated in 15 and 30% sucrose, and then embedded in OCT medium. Tissues were sectioned at 10 μm thickness. The surface preparations or cryosections were permeabilized in 0.25% Triton X-100 solution at RT for half an hour, and then blocked in 10% goat serum at RT for an hour. Incubation with primary antibody was performed at 4°C overnight. The primary antibodies used in this step were as follows: rabbit anti-NFAT3 polyclonal antibody (1:400, Abcam, ab3447) (Kohno et al., 2020), mouse anti-FasL monoclonal antibody (1:200, Santa Cruz, sc19988) (Lu et al., 2012), rabbit anti-P-MLKL (1:200, Abcam, ab196436) (Chen et al., 2022), rabbit anti-cleavage caspase 3 (1:200, Beyotime, AC033) (Han et al., 2022), mouse anti-Fas (1:100, santa cruz, sc-74540) (Wang et al., 2023), and rabbit anti-cleavage caspase 8 (1:50, CST 8592) (Zheng et al., 2014). After rinsing three times, specimens were incubated with Alexa Fluor 488-labeled goat anti-rabbit IgG or Alexa Fluor 647-labeled goat anti-mouse IgG absent from light for one hour at RT. After rinsing, surface preparations were counterstained with Alexa Fluor 555 phalloidin (CST, #8953) absent from light for 15 min at RT. After the final wash in PBS, the epithelia were dissected into three segments (apex, middle and base) and then mounted on slides using Antifade Mounting Medium with Hoechst 33342 (Beyotime, P0133). For cryosections, the specimens were rinsed in PBS to remove residual secondary antibodies and then mounted with antifade mounting medium containing Hoechst 33342 directly. Control samples were routinely prepared in parallel without primary antibodies. Immunofluorescence confocal images were obtained with an LSM 980 laser confocal microscope (Carl Zeiss).

### 2.6 Quantification of condensed, swollen, and missing OHCs on surface preparations

Two hours after the noise exposure, the mice were sacrificed to obtain surface preparations. Hoechst 33342 or propidium iodide (PI) plus phalloidin were used to label OHC nuclei and cytoskeleton. The images of the lower basal turn of the cochlear epithelium (4.5–5.5 μm from the apex) were captured using a 63X magnification lens to quantify condensed, swollen (diameter ≥ 7 μm) and missing OHCs according to protocols (Zheng et al., 2014). Condensed OHC nuclei were identified as apoptotic cells, and swollen nuclei were recognized as necrotic cells (Bohne et al., 2007). The areas with phalloidin stained cytoskeleton but without Hoechst 33342 labeled nuclei were identified as missing OHCs.

### 2.7 Hair cell counts of surface preparations

Two weeks after the noise exposure, animals were euthanized to prepare cochlear surface preparations. Hoechst 33342 and phalloidin were utilized for labeling OHCs nuclei and cellular structure. The whole length of the cochlear sensory epithelium was captured by Zeiss confocal microscope using its automatic image stitching mode and a 40X lens. In order to get the complete structure of cochlear hair cells, multi-layer cross-sections of hair cells from the bottom to the top were also captured. OHCs with a cellular cytoskeleton but no nucleus were considered to be lost OHCs (Supplementary Figure S2). IHCs and OHCs were counted from the apex to the base of the cochlear epithelium, and the percentage of hair cell loss was plotted every 0.5 mm length of the cochlear epithelium to draw a cytocochleogram.

### 2.8 Quantitative analysis of immunofluorescence intensity of OHCs from surface preparations

To ensure the comparability of immunofluorescence signals from different experimental groups, samples from different groups were processed in parallel with the same reagents and antibody solutions. Zeiss ZEN Blue image processing software was utilized to measure fluorescence intensities from original confocal images with a 63X magnification. Also, identical microscope parameters (such as laser intensity, pinhole diameter, and gain settings) were used among different slides. To determine the fluorescence intensity of target proteins, we selected the upper basal turn of the cochlear epithelium (generally 0.12 mm long and contains 60 OHCs) to calculate the mean fluorescence intensity of these OHCs (Zheng et al., 2014). The background fluorescence intensity of the adjacent non-cell region was then subtracted. To determine the nuclear translocation of NFAT3 in individual OHCs, OHCs were classified as “nuclear NFAT3 positive” when the fluorescence intensity of nuclear NFAT3 was twofold higher than the mean fluorescence intensity of nuclear NFAT3 in the control group.

### 2.9 Western blotting

After mice were sacrificed, temporal bones were removed immediately and dissected in ice-cold PBS to obtain cochleae. Two cochleae from one mouse were pooled together to create a single sample. Total protein was extracted using SDS lysis buffer (containing 50 mM Tris, 1% SDS, sodium pyrophosphate, β-glycerophosphate, sodium orthovanadate, sodium fluoride, EDTA, and leupeptin, Beyotime, P0013G) supplemented with a mixture of phosphatase and protease inhibitors (Beyotime, P1051). Cytosolic and nuclear proteins were extracted using a nuclear and cytoplasmic protein extraction kit (Beyotime, China) based on the manufacturer’s instructions. The BCA protein assay kit (Beyotime, China) was employed to quantify the protein concentration. Proteins (20 μg for each group) were separated by SDS-PAGE gel electrophoresis and transferred to PVDF membranes. After being blocked in 5% non-fat milk/TBST, the membranes were probed with primary antibodies against NFAT3 (1:1000, Abcam, ab3447), FasL (1:1000, Santa Cruz, sc19988), cleavage caspase 3 (1:1000, Beyotime, AC033), and MLKL (1:1000, Beyotime, AF5231) overnight on a shaker at 4°C. To detect P-MLKL, the membranes were blocked in 5% BSA/TBST, and then incubated with anti-P-MLKL antibody (1:1000, Abcam, ab196436) overnight on a shaker at 4°C. After rinsing with TBST to remove residual unbound primary antibodies, horseradish peroxidase-conjugated secondary antibodies (goat anti-rabbit or goat anti-mouse, 1:10000, Beyotime) diluted in 5% BSA/TBST were added and incubated at RT for 1 h. After rinsing, the membranes were visualized using the Western Blotting luminol reagent (Santa Cruz, sc-2048).

### 2.10 Delivery of siRNA via the round window

Intra-tympanic administration of siFasL (Invitrogen, #AM16708) or scrambled RNA control (siControl, Invitrogen, #4,390,843) was locally performed onto the round window niche (RWN), as described previously (Wu et al., 2022; Fan et al., 2023). After shaving and disinfecting the post-auricular region of the ear, a retroauricular incision was performed to access the temporal bone. We used a 30-G needle to create a hole on the otic bulla. A micro-injection was inserted into the hole above the RWN to slowly deliver 10 μL (0.6 μg per ear) of pre-designed siRNA. Filled the hole with muscle and tissue adhesive, and sutured the skin and subcutaneous tissue. The mouse was kept in the surgical position under anesthesia for 1 h to facilitate drug absorption.

### 2.11 Statistical analysis

Continuous data were presented as mean ± standard deviation (SD), and categorical data were presented as percentages. The difference between two independent groups was compared using Student’s *t*-test. *p* < 0.05 was considered as statistically significant. Statistical analyses were performed by SPSS (version 20.0) and GraphPad Prism 8.

## 3 Results

### 3.1 The expression pattern of NFAT3 and FasL in the cochlea of normal C57BL/6 mice

**Figure 1:**
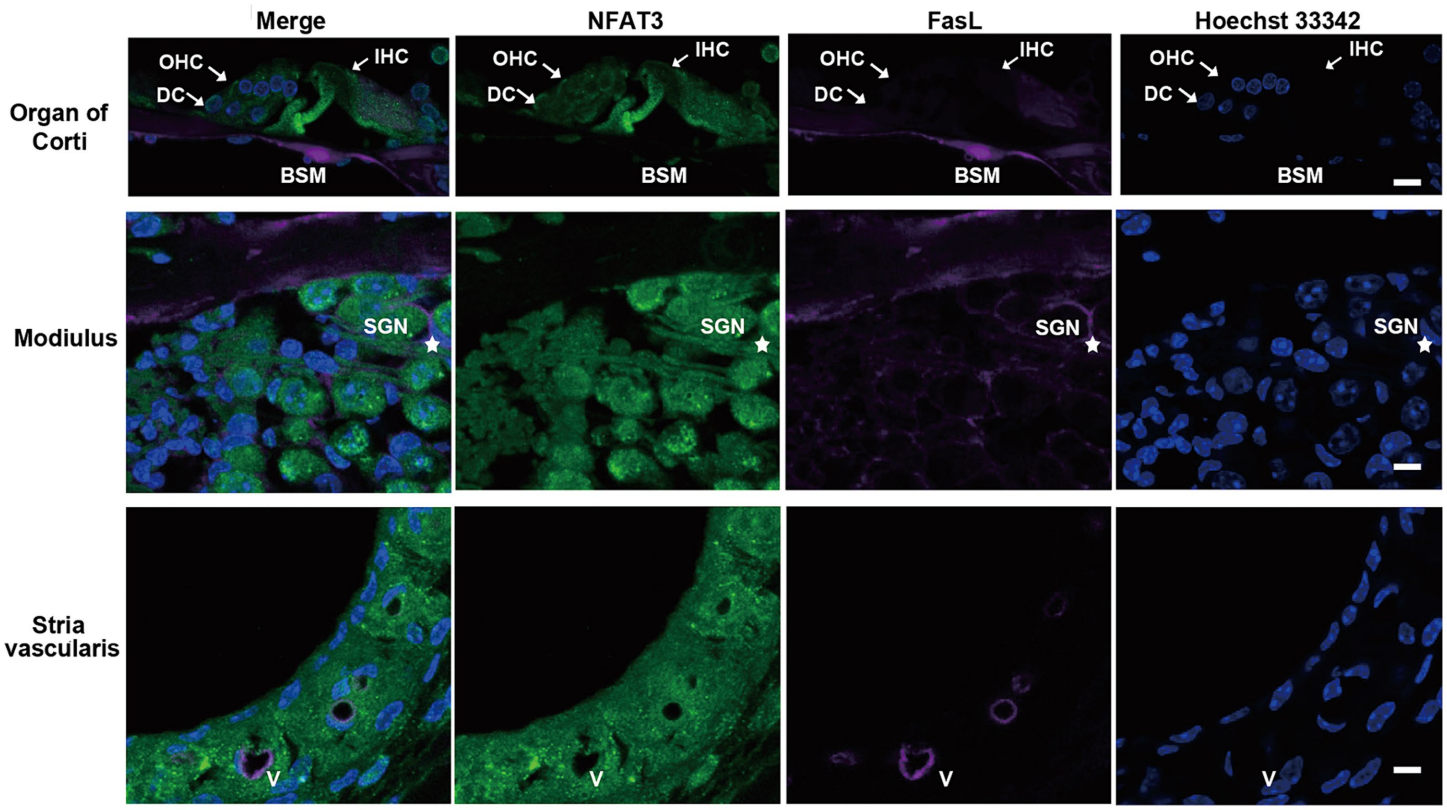
The expression pattern of NFAT3 and FasL in normal cochlea from C57BL/6 J mice. Representative images show immunofluorescence staining of cochlear frozen sections for NFAT3, FasL and Hoechst 33342. Abundant dotted NFAT3 fluorescence was observed in inner hair cells (IHCs), outer hair cells (OHCs), Deiters supporting cells (DCs), spiral ganglion neurons (SGNs), and the stria vascularis. FasL fluorescence staining was only observed on the basement membrane (BSM) of the organ of Corti, the myelin sheath enwrapping SGNs (indicated by an asterisk), and vessel (V) walls of the stria vascularis. *N* = 5 mice; scale bar = 10 μm.

**Figure 2:**
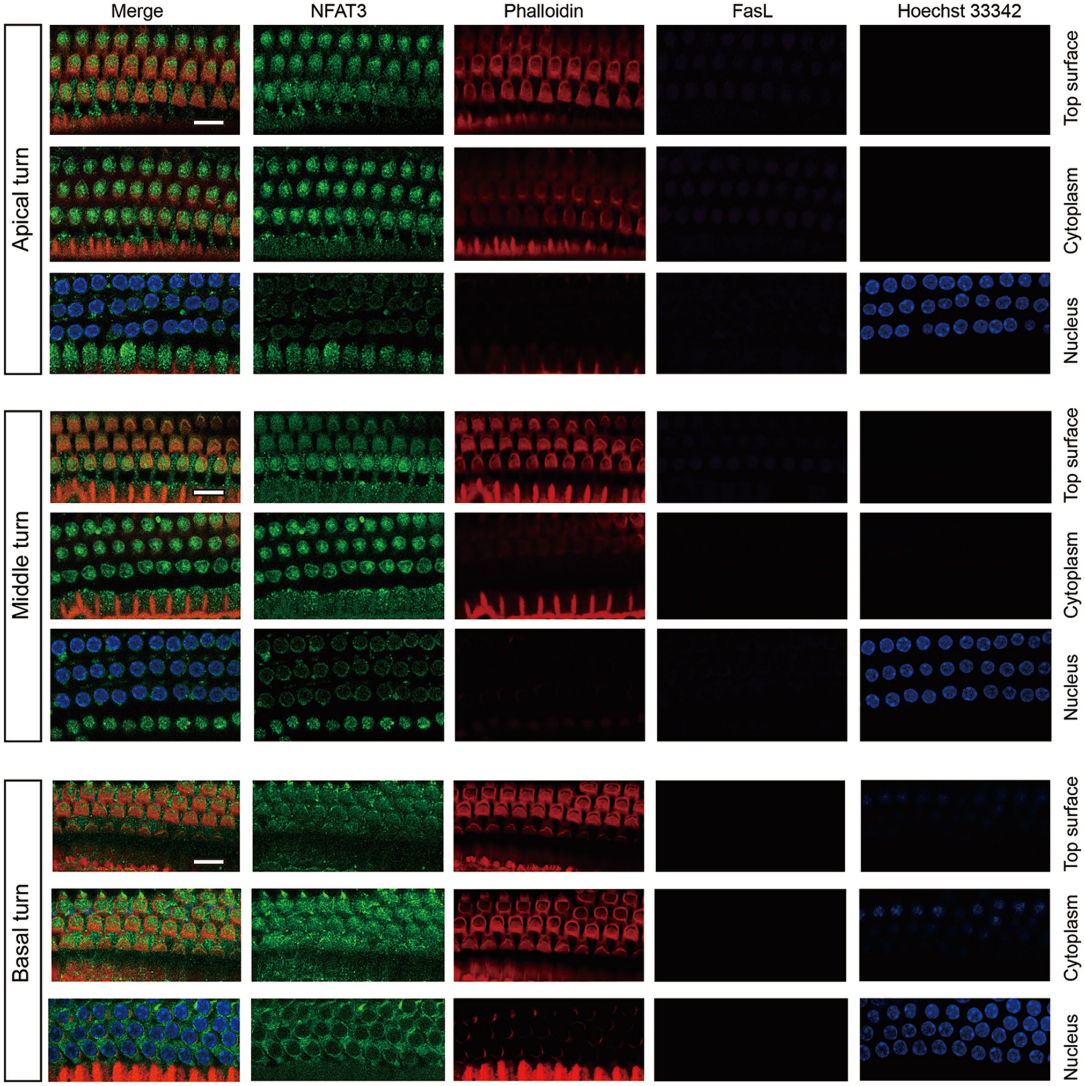
The expression pattern of NFAT3 and FasL in normal OHCs from C57BL/6 J mice. NFAT3 labeling was primarily observed in the cytoplasm of OHCs, with negligible signal detected in the nuclei. The fluorescence labeling of FasL could not be identified in the OHCs throughout the entirety of the cochlear epithelium. Phalloidin was used to label the cytoskeleton of cochlear hair cells, and Hoechst 33342 was utilized for nuclear labeling. Scale bar = 10 μm; *N* = 5 mice.

We first examined the expression pattern of NFAT3 and FasL in the cochlea of 6-week-old C57BL/6 mice using immunofluorescence staining. The NFAT3 antibody we employed in our experiments recognized both phosphorylated and unphosphorylated forms of NFAT3. Frozen sections were utilized to analyze the expression, revealing widespread distribution of NFAT3 in the organ of Corti (including IHCs, OHCs, and supporting cells), spiral ganglion, and stria vascularis (Figure 1). In the normal cochlea, only a few cells exhibited FasL expression. Weak staining of FasL was observed in the basement membrane of the organ of Corti, the vascular wall of the stria vascularis, and the myelin sheath surrounding the spiral ganglion neuron. However, frozen sections only provide a longitudinal section representation of a limited number of sensory hair cells, which may not fully reflect the expression pattern of the target proteins. To obtain a more comprehensive view, we further investigated the immunofluorescence staining of the entire cochlear epithelium (Figure 2). Past research has indicated that noise exposure primarily induces the death of OHCs in the cochlea (Xiong et al., 2019). As a result, our study specifically focused on the biological processes occurring in OHCs. Abundant dotted NFAT3 fluorescent labeling was observed in the cytoplasm of OHCs, while almost no NFAT3 staining was detected in the nucleus. Moreover, no FasL staining was observed throughout the whole length of the cochlear epithelium. Together, these findings indicate that the NFAT3/FasL signaling pathway is inactive in normal OHCs, providing essential knowledge for our subsequent experiments.

### 3.2 Noise induces permanent auditory threshold shift and loss of OHCs in C57BL/6 mice

**Figure 3:**
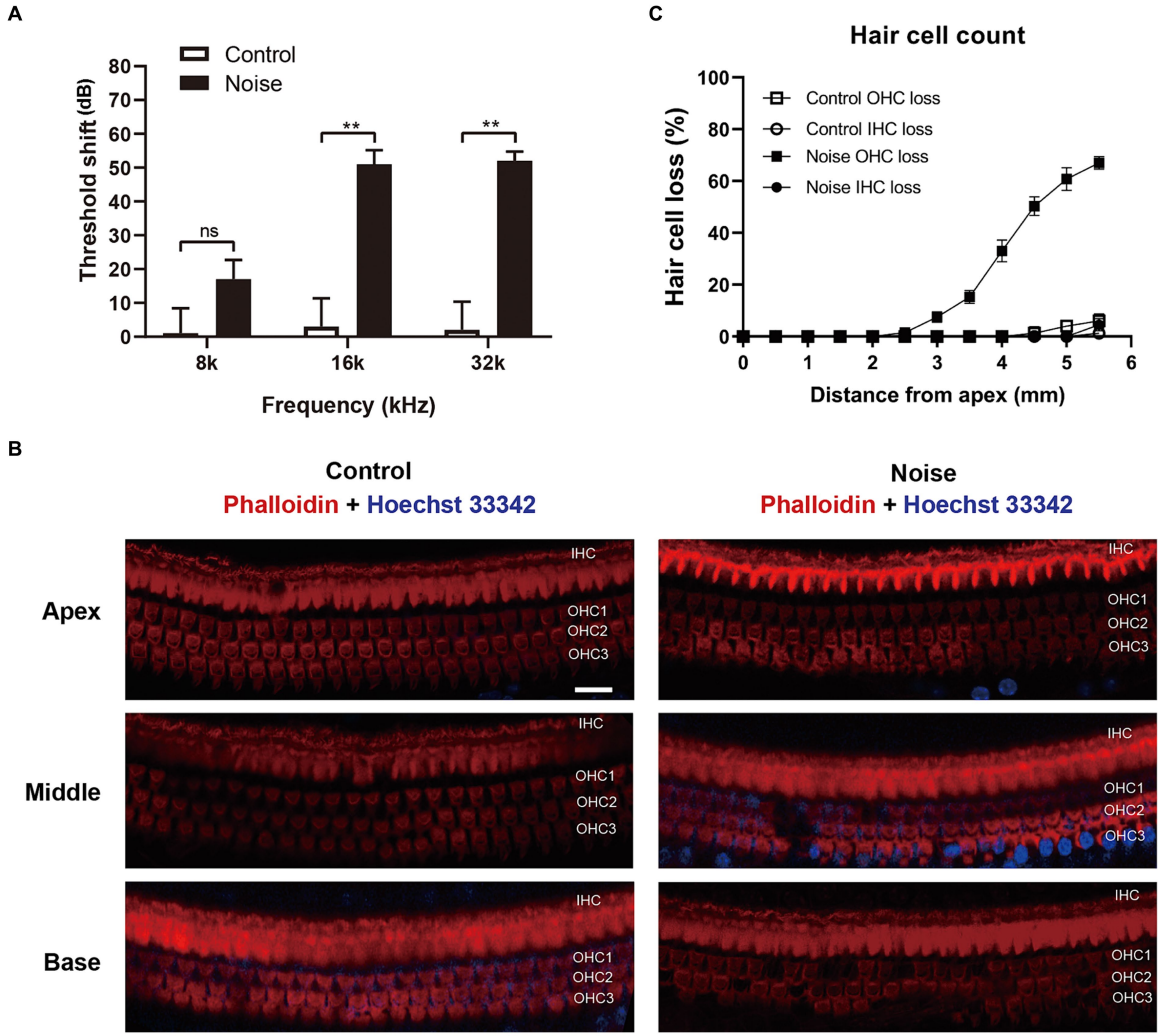
Noise exposure causes permanent auditory threshold shift and loss of OHCs in C57BL/6 J mice. **(A)** ABR measurement showed that exposure of mice to 108 dB white noise for 2 h induced permanent threshold shift at 16 kHz and 32 kHz. **(B)** Immunofluorescence staining for phalloidin and Hoechst 33342 of the full-length cochlea showed that there was significant loss of OHCs in the basal turn of cochlear epithelia. OHC 1, 2, 3 and IHC represent three rows of OHCs and one row of IHCs. **(C)** Quantification analysis of hair cell loss in the whole cochlear epithelium 2 weeks after noise exposure. Scale bar = 20 μm; ^**^p < 0.01, ns: not significant; *N* = 5 mice.

Previous studies have demonstrated that exposure of CBA/J mice to 98 dB SPL broadband noise for 2 h leaded to permanent auditory threshold shift and loss of OHCs, while IHCs remained intact (Zheng et al., 2014). The C57BL/6 mouse strain, which carries the ahl mutation in the Cdh23 gene resulting in accelerated hearing loss, is commonly used in hearing research (Fu et al., 2021). However, there is limited information available regarding the characteristics of sensory hair cell loss in C57BL/6 mice after noise exposure. In this study, 6-week-old male C57BL/6 mice were exposed to 108 dB white noise for 2 h to induce permanent hearing loss. As shown in Figure 3A, mice presented with significant ABR threshold shift of ~50 dB at 16 kHz and 32 kHz 2 weeks after noise trauma. The loss of sensory hair cells was also examined (Figures 3B,C). Two weeks after noise exposure, the loss of OHCs initiated at a distance of 2.5 mm from the apex and increased progressively towards the base of the cochlear epithelium. Meanwhile, there was minimal noticeable loss of IHCs throughout the cochlear epithelium. These findings indicated that the hearing loss observed in C57BL/6 mice following exposure to 108 dB white noise was primarily attributed to the loss of OHCs.

### 3.3 Traumatic noise triggers FasL-related apoptosis and necroptosis in OHCs

**Figure 4:**
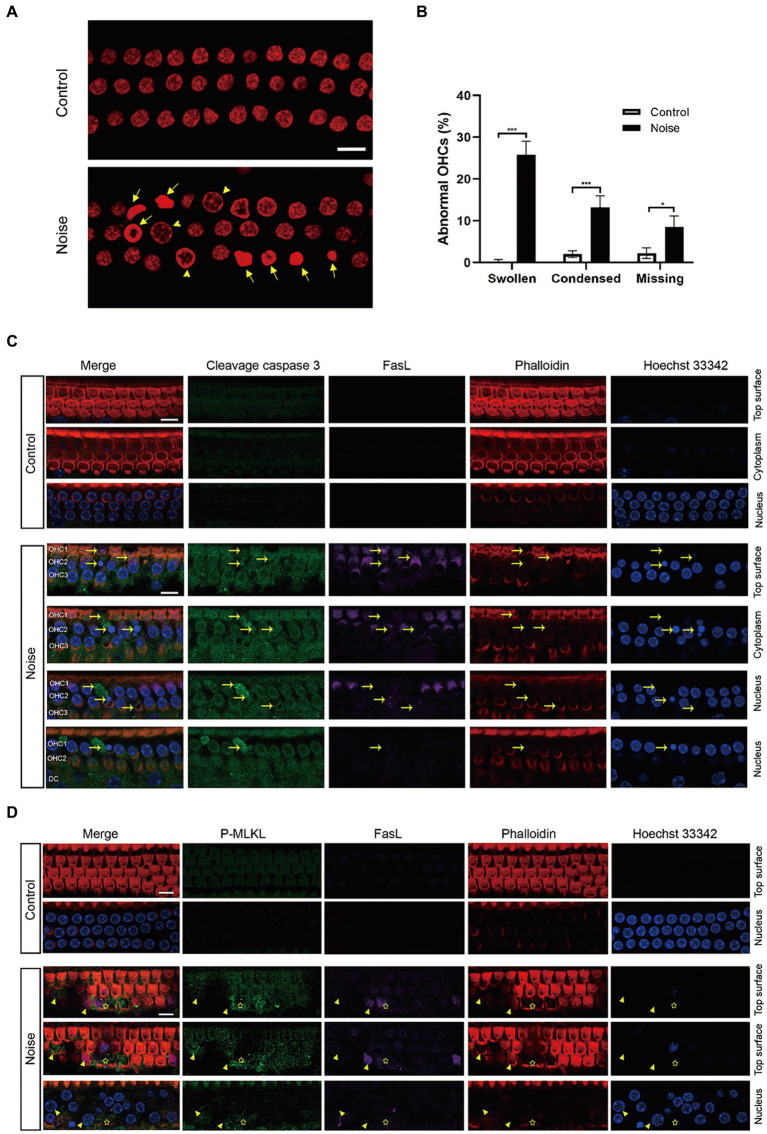
Noise induces FasL-related apoptosis and necroptosis in OHCs after noise exposure. **(A)** Representative images of propidium iodide staining of OHCs in the basal turn of the cochlea, collected 2 h after noise exposure. Arrows indicate apoptotic nuclei (condensed), while triangles indicate necrotic nuclei (swollen with diameter ≥ 7 μm). **(B)** Quantitative analysis of abnormal OHCs nuclei in the basal turn of the cochlea after noise trauma. **(C)** Immunofluorescence staining of the basal turn of cochlear epithelia showed that noise upregulated the expression of cleavage caspase 3 and FasL in OHCs. Specific enhanced cleavage caspase 3 and FasL fluorescence signals were observed in apoptotic OHCs (arrow). Due to the oblique arrangement of OHCs, the three rows of OHCs were not on the same plane. The lower panel shows cross sections of OHC1 from the top to the nucleus plane. OHC 1, 2, 3 and DC represent three rows of OHCs and Deiters supporting cell. **(D)** Immunofluorescence staining of the basal turn of cochlear epithelia showed that noise upregulated the expression of P-MLKL and FasL in OHCs, and specific enhanced P-MLKL and FasL fluorescence signals were observed in necrotic OHCs (swollen nuclei, indicated by triangle) and regions where OHCs disintegrated (asterisk). After noise trauma, P-MLKL and FasL were mainly expressed on the top surface of OHCs. Scale bar = 10 μm; ^*^p < 0.05; ^***^p < 0.001; *N* = 5 mice.

To identify the type of hair cell death induced by acoustic trauma, we analyzed the morphology of the nuclei in OHCs. The basal turn of the cochlear epithelia was selected for observation 2 h after noise exposure, as this period displayed the most significant death of OHCs. Following this, the OHCs quickly disintegrated, rendering them invisible to observation. As shown in Figures 4A,B, there were significantly increased apoptotic OHCs (condensed nuclei), necrotic OHCs (swollen nuclei with diameter ≥ 7 μm), and missing OHCs 2 h after noise exposure.

The general morphological features of necroptosis include nuclear and organelle swelling, as well as plasma membrane rupture (Zhang et al., 2018). Zheng’s et al. (2014) study previously demonstrated that noise exposure induces both apoptosis, characterized by condensed nuclei, and necrosis, identified by swollen nuclei, in OHCs. Furthermore, the form of necrosis in OHCs was identified as necroptosis, which could be inhibited by Nec-1 (Zheng et al., 2014). The phosphorylated mixed lineage kinase domain-like protein (P-MLKL) acts as the effector protein in the necroptosis signaling pathway. Our results revealed that noise exposure increased the expression of cleavage caspase 3 and P-MLKL in OHCs (Figures 4C,D). The fluorescence signals of cleavage caspase 3 and P-MLKL were specifically enhanced in apoptotic and necrotic OHCs. Furthermore, noise exposure upregulated FasL expression in OHCs, particularly in apoptotic and necrotic OHCs. The observed spatial association between FasL expression, morphological changes in apoptotic and necrotic nuclei, and the activation of caspase 3 and MLKL suggested that FasL activity was functionally connected to both apoptosis and necroptosis in OHCs following noise exposure.

**Figure 5:**
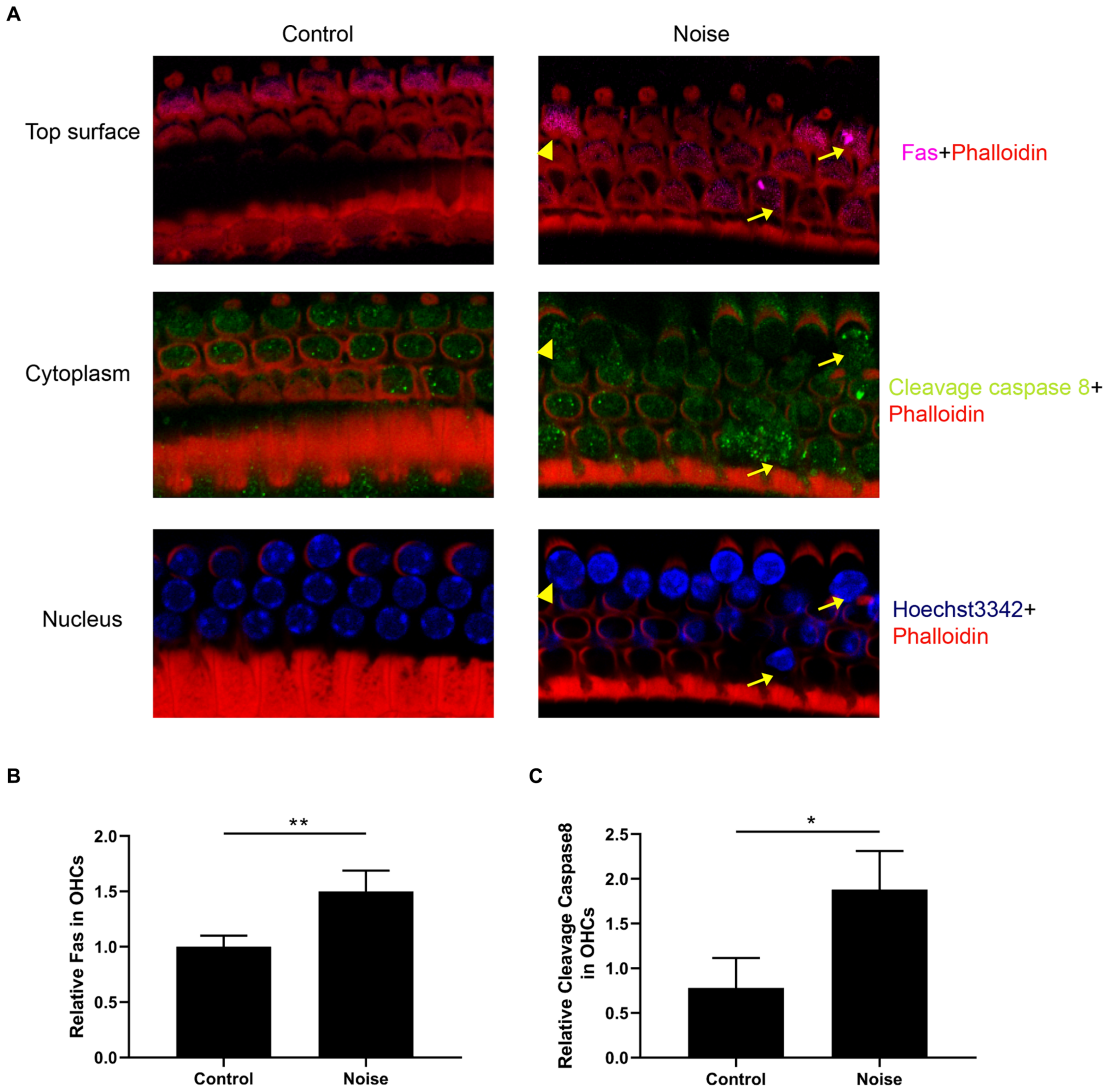
Noise induces upregulation of Fas receptor and cleavage caspase 8 expression in OHCs. **(A)** Representative immunofluorescence staining for Fas and cleavage caspase 8 in OHCs from the basal turn of the cochlear epithelia 2 h after noise exposure. Arrows indicate apoptotic OHCs, and triangles indicate necrotic OHCs. Semi-quantitative analysis of the fluorescence intensity of Fas **(B)** and cleavage caspase 8 **(C)** in the OHCs from the basal turn of the cochlea. N = 5 mice, ^*^p < 0.05, ^**^p < 0.01.

We further investigated the expression of the Fas receptor and cleavage caspase 8 following noise exposure. As shown in Figure 5, normal OHCs exhibited Fas receptor expression, predominantly localized within the top surface. After noise exposure, there was a significant upregulation of Fas expression within OHCs, particularly pronounced in apoptotic and necrotic OHCs. Furthermore, noise exposure significantly elevated the expression level of cleavage caspase 8 in OHCs, particularly intensifying in those undergoing apoptosis.

**Figure 6:**
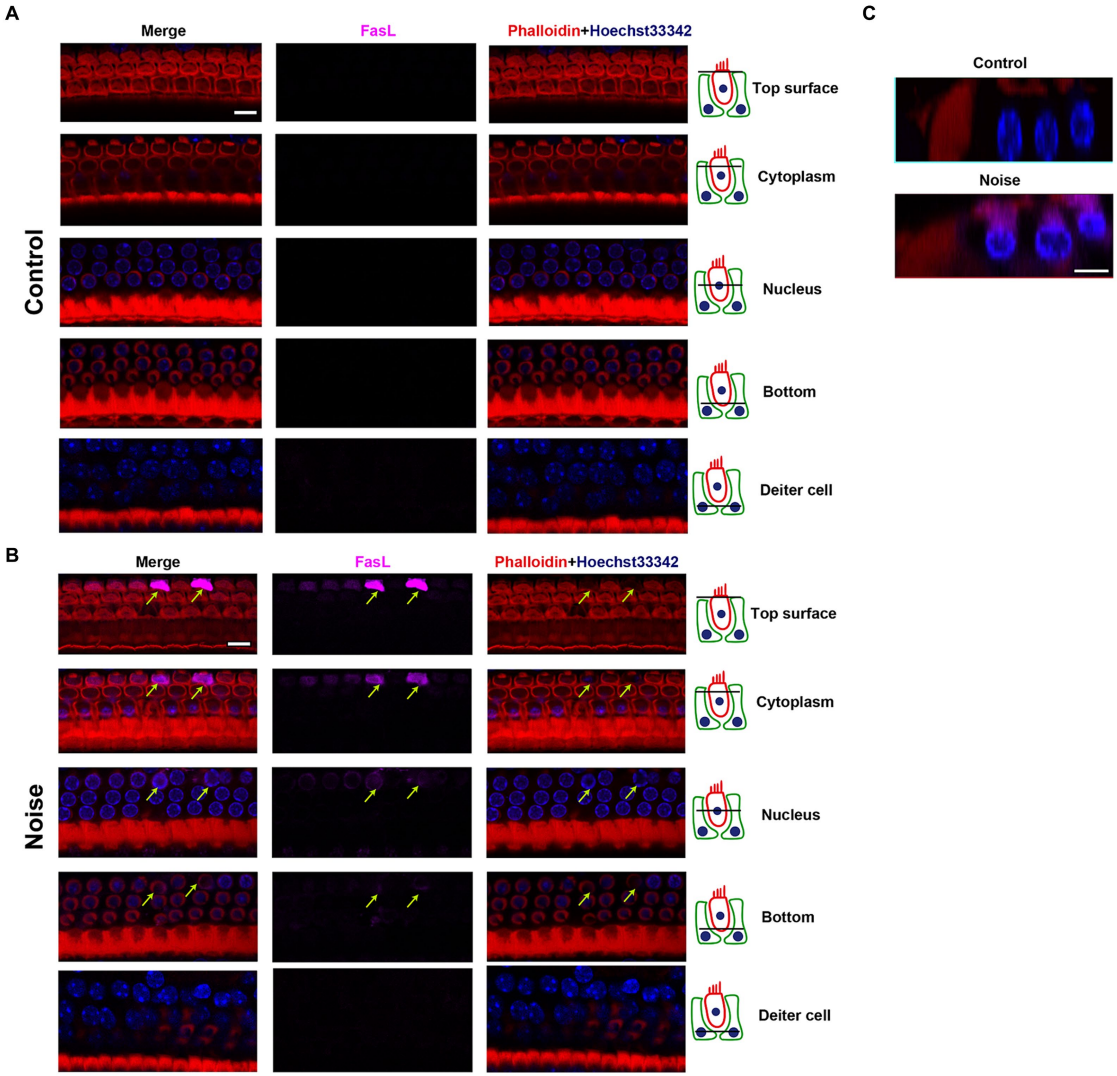
Expression pattern of FasL in OHCs after noise exposure. Representative multiple immunofluorescence staining for FasL/Phalloidin/Hoechst33342 in the basal turn of cochlear epithelia from control mice **(A)** and mice 2 h after noise exposure **(B)**. The figures display different cross sections, ranging from the top of OHCs to the supporting cell (Deiters cell) level. In OHCs from control mice, FasL staining was not observed. After noise exposure, there was specific enhanced FasL staining in the apoptotic OHCs (indicated by arrows). The strongest FasL staining was observed on the top surface of OHCs near the cuticular plate, while the weakest staining was seen on the bottom of OHCs. No FasL staining was observed at the supporting cell level. **(C)** The cross-sectional view of OHCs. Scale bar = 10 μm.

**Figure 7:**
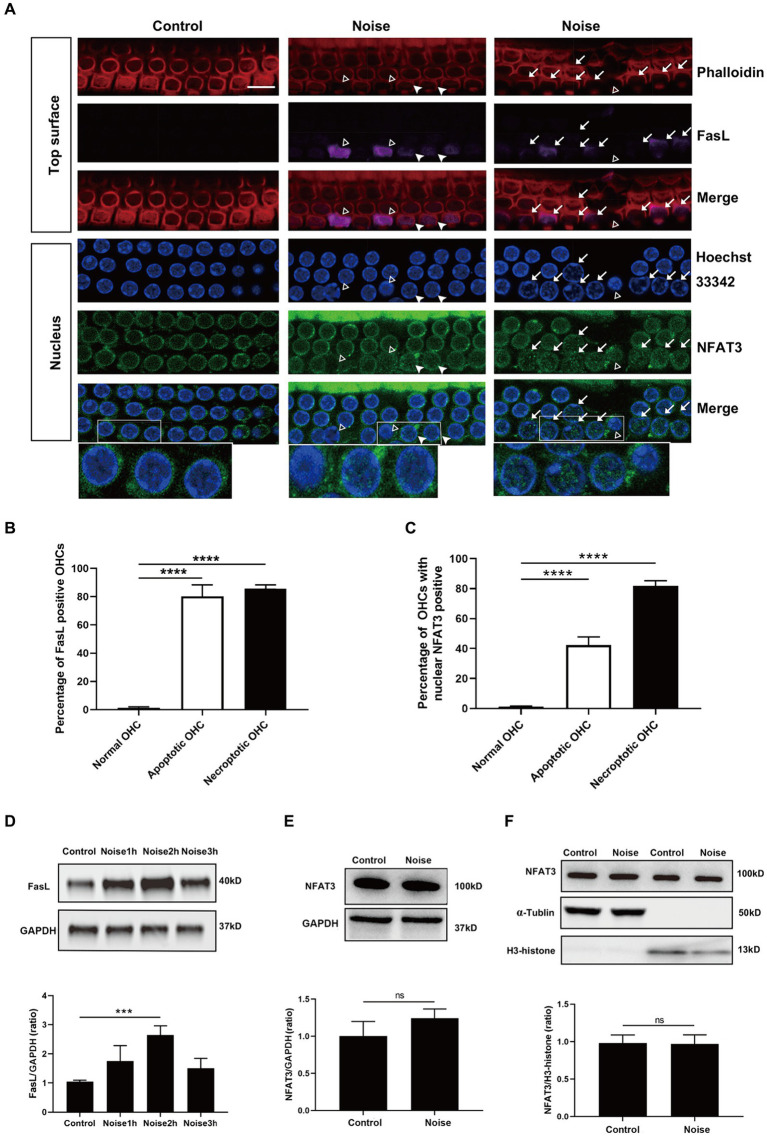
Noise-induced apoptosis and necroptosis of OHCs are related to NFAT3 nuclear translocation and FasL expression. **(A)** Immunofluorescence staining of OHCs in the basal turn of cochlear epithelia 2 h after noise exposure, revealed enhanced expression of FasL and nuclear accumulation of NFAT3 in the apoptotic (marked with open triangles) and necroptotic (marked with arrows) OHCs. White triangles indicate cells potentially in the early stage of apoptosis/necroptosis, characterized by significant nuclear accumulation of NFAT3 and relatively weak FasL staining without pronounced nuclear morphological changes. Bottom insets display local magnifications. Quantitative analysis of the percentage of FasL-positive cells **(B)** and nuclear NFAT3-positive cells **(C)** in normal OHCs, as well as in apoptotic and necroptotic OHCs. **(D)** Representative western blot analysis of FasL in whole cochlear homogenates from control mice and mice after noise exposure for the indicated periods. **(E)** Representative western blot analysis of NFAT3 in whole cochlear homogenates 2 h after noise exposure. **(F)** Representative western blot analysis of NFAT3 in cytoplasmic and nuclear fractions from the cochlea 2 h after noise exposure. α-Tubulin and H3-histone were used as loading controls for cytoplasmic and nuclear fractions, respectively. Scale bar = 10 μm; ^***^p < 0.001, ^****^p < 0.0001, ns: not significant; N = 5 mice.

Figure 6 showed the expression pattern of FasL in OHCs after noise exposure. FasL was mainly expressed on the top surface of OHCs. The expression of FasL was weak at the bottom of OHCs and the supporting cell level (Deiters cell). There was a specific enhancement of FasL staining in the apoptotic and necroptotic OHCs (see also in Figure 7A and Supplementary Figure S3), rather than adjacent cells or supporting cells. This indicated that the death signal of FasL came from apoptotic and necroptotic OHCs themselves, rather than adjacent OHCs or supporting cells. Combining the above findings indicating that noise induced FasL-related apoptosis and necroptosis in OHCs, these results suggested that OHCs committed autonomous apoptosis and necroptosis via the FasL/Fas system during NIHL.

### 3.4 Noise-induced apoptosis and necroptosis of OHCs are associated with NFAT3 nuclear translocation and FasL expression

The above preliminary results suggested that the NFAT3/FasL signaling pathway was silent in normal OHCs. Here, we investigated the potential link between the activation of NFAT3/FasL signaling axis and OHCs death after noise trauma. We examined the OHCs of basal turns 2 h after noise exposure. The results showed that the apoptosis and necroptosis of OHCs were significantly related to NFAT3 nuclear translocation and FasL expression (Figure 7A and Supplementary Figure S3). The majority of the necroptotic and apoptotic OHCs were labeled with FasL staining on their top surface (Figure 7B). About 80% of the necroptotic OHCs and 40% of the apoptotic OHCs presented with punctate NFAT3 fluorescence in their nuclei (Figure 7C). Furthermore, NFAT3 nuclear translocation and FasL expression were colocalized in the apoptotic/necroptotic OHCs, suggesting that these events were functionally associated.

Apart from the activation of NFAT3/FasL signaling in specific cell populations observed by immunofluorescence staining, the expression of NFAT3 and FasL in the cochlea might also change in response to noise exposure. To test the possibility, western blot analyses of whole cochlear homogenates, cytoplasmic and nuclear fractions were performed. As shown in Figure 7D, the expression level of FasL in the cochlea gradually increased by about 1.5 times at 2 h after noise exposure, and then decreased. However, the total NFAT3 level (Figure 7E) and the nuclear NFAT3 level (Figure 7F) were not changed after noise exposure, indicating that there was no obvious nuclear translocation of NFAT3 in the cochlea. Considering that there were many other cell types in the cochlea besides sensory hair cells, and the widespread distribution of NFAT3 in the cochlea, it was not surprising that western blot could not detect changes of NFAT3 in the whole cochlea.

### 3.5 Inhibition of NFAT3 nuclear translocation with FK506 and 11R-vivit diminishes FasL expression, apoptosis and necroptosis of OHCs, and protects against NIHL

**Figure 8:**
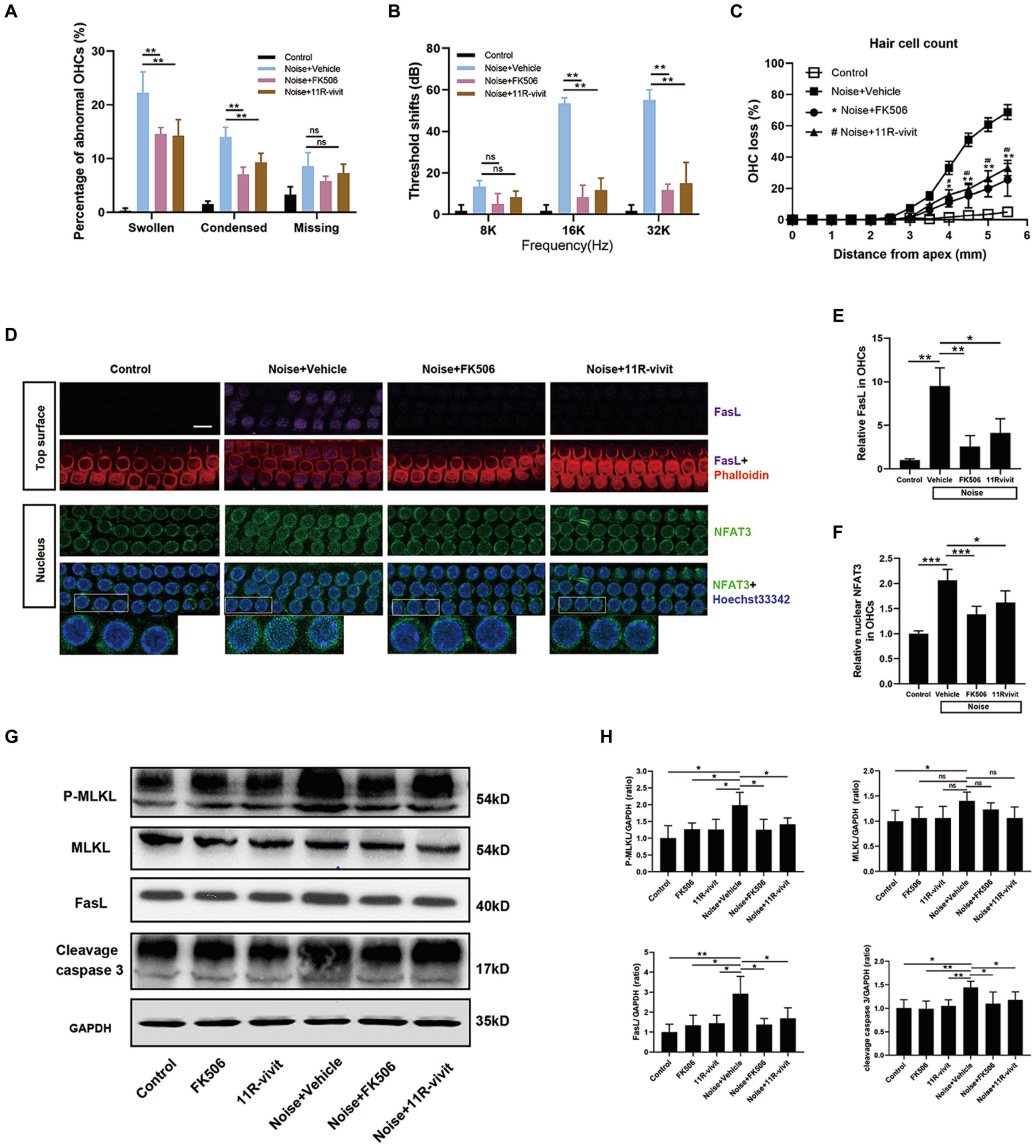
Administration of either FK506 or 11R-vivit inhibits NFAT3 nuclear translocation, suppresses FasL expression, attenuates apoptosis and necroptosis in OHCs, and protects against NIHL. **(A)** Quantification analysis of abnormal OHCs nuclei (abnormal OHCs/total OHCs) in the basal turn of cochlear epithelia 2 h after noise exposure. **(B)** The auditory threshold shift was measured by ABR test 2 weeks after noise exposure. **(C)** Quantification analysis of OHCs loss in the entire cochlea 2 weeks after noise exposure. **(D)** Multiple immunofluorescence staining for FasL/NFAT3/phalloidin/Hoechst33342 was performed on the basal turn of cochlear epithelia immediately after noise exposure. Bottom insets display local magnifications. Semi-quantification analysis of the immunofluorescence intensity reveals that FK506 and 11R-vivit significantly reduced noise-induced FasL expression **(E)** and NFAT3 nuclear accumulation **(F)** and in OHCs. N = 5 mice. **(G)** Western blot analysis of cochlear homogenates 2 h after noise exposure, measuring the expression levels of P-MLKL, MLKL, FasL and cleavage caspase 3. **(H)** Semi-quantitative analyses of the relative band densities of P-MLKL, MLKL, FasL, and cleavage caspase 3 compared with GAPDH. Scale bar = 10 μm; ^*, #^*p* < 0.05, ^**, ##^*p* < 0.01, ^***^p < 0.001; ns: not significant; *N* = 5 mice.

We tried to block NFAT3 activation using pharmacological inhibitors to determine whether the activation of NFAT3 was responsible for NIHL. FK506, an extensively used immunosuppressive agent, non-specifically inhibits calcineurin activity by binding its catalytic subunit. 11R-vivit is a cell-permeable short peptide that specifically inhibits the activation of NFAT without affecting other substrates of calcineurin such as NFκB and TNF-β (Aramburu et al., 1999). In the present study, quantification analysis of abnormal OHCs nuclei showed that both agents significantly reduced the number of apoptotic and necroptotic OHCs nuclei 2 h after noise exposure (Figure 8A). ABR measurements revealed that FK506 and 11R-vivit significantly attenuated NIHL at 16 kHz and 32 kHz 2 weeks after noise exposure (Figure 8B). Quantification analysis of the loss of OHCs 2 weeks after noise exposure indicated that FK506 and 11R-vivit significantly reduced the noise-induced loss of OHCs (Figure 8C).

We selected the basal turn of the cochlear epithelium immediately after noise exposure to study the molecular biology processes of OHCs, at which time there was no obvious cell death and disintegration. Immunofluorescence staining showed an upregulation of nuclear NFAT3 and FasL in OHCs immediately after noise exposure, and the expression was relatively uniform (Figure 8D). This was different from the specific expression pattern of FasL and nuclear NFAT3 in the damaged OHCs 2 h after noise exposure. This suggested that the sustained activation of NFAT3/FasL signaling in OHCs might trigger cell death pathways. FK506 and 11R-vivit significantly blocked the NFAT3 nuclear translocation and the FasL up-regulation (Figures 8E,F). Besides, western blot analyses were performed to detect the key molecules of programmed cell death pathways (Figure 8G). The results showed that FK506 and 11R-vivit significantly reduced noise-induced up-regulation of FasL, P-MLKL, and cleavage caspase 3 in the cochlea (Figure 8H). To sum up, inhibition of NFAT3 transcription activity reduced the expression of FasL and alleviated apoptosis and necroptosis of OHCs, thereby protecting against NIHL.

### 3.6 FasL silencing alleviates apoptosis and necroptosis of OHCs, and protect against NIHL

**Figure 9:**
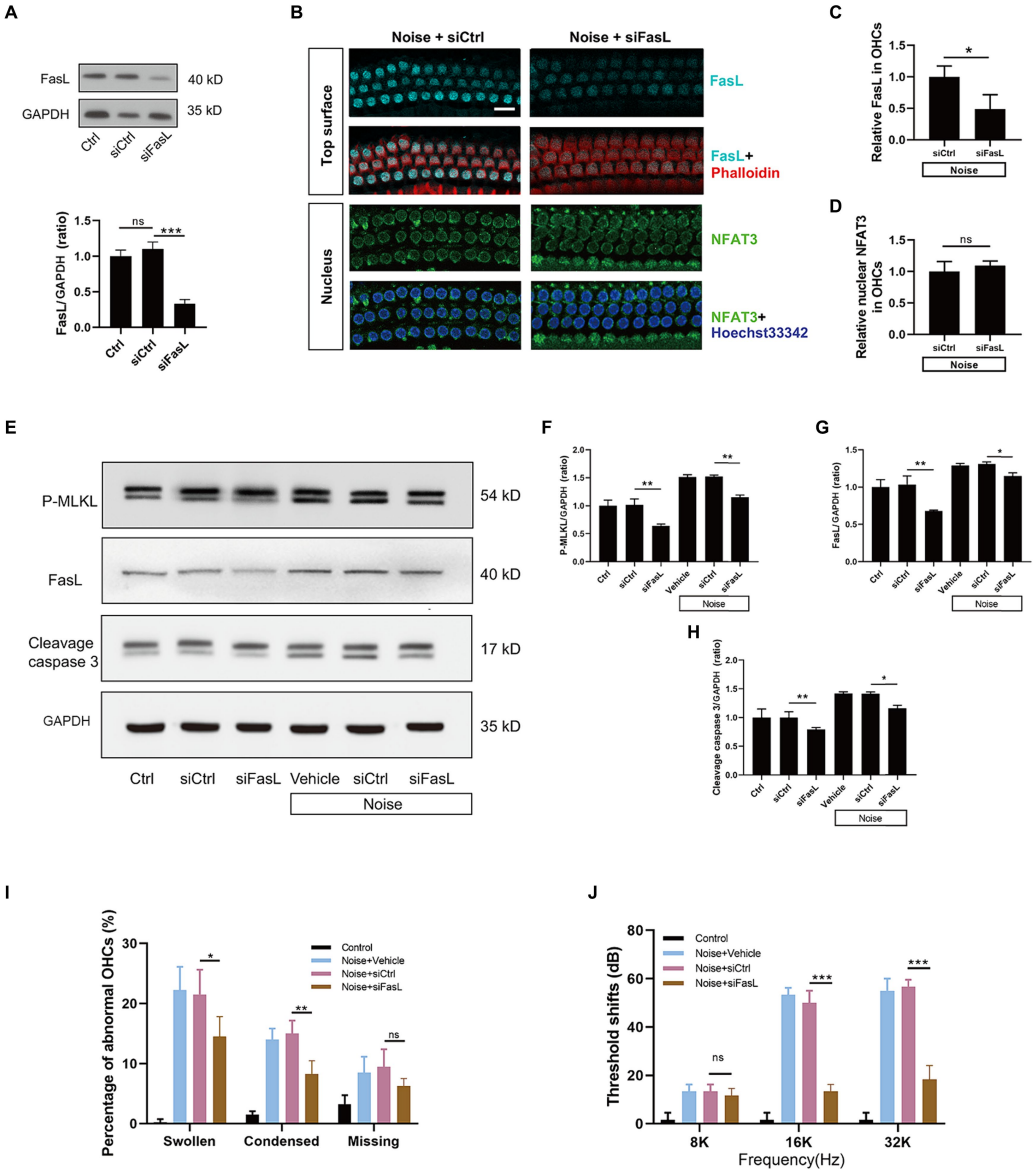
Administration of siFasL via RWN mitigates noise-induced apoptosis and necroptosis in OHCs and ameliorates NIHL. **(A)** Western blot analysis of cochlear homogenates showed that siFasL treatment reduced FasL expression by approximately 70% in the cochlea. **(B)** Immunofluorescence staining for FasL (cyan) and NFAT3 (green) in the basal turn of cochlear epithelia immediately after noise exposure. Semi-quantitative analysis of immunofluorescence intensity showed that siFasL significantly reduced FasL expression by approximately 50% in OHCs **(C)**, with no obivious effect on the nuclear NFAT3 expression **(D)**. *N* = 5 mice. **(E)** Western blot analysis of cochlear homogenates 2 h after noise exposure for P-MLKL, cleavage caspase 3, and FasL. Band densities of P-MLKL **(F)**, cleavage caspase 3 **(G)**, and FasL **(H)** were quantified, with GAPDH serving as the loading control. **(I)** Quantitative analysis of abnormal OHCs nuclei in the basal turn of cochlear epithelia 2 h after noise exposure. **(J)** The auditory threshold shift was measured by ABR 2 weeks after noise exposure. Data are represented by mean ± SD; scale bar = 10 μm; ^*^p < 0.05, ^**^p < 0.01, ^***^p < 0.001, ns: not significant; *N* = 5 mice.

To investigate the role of FasL in OHCs death, siFasL or scrambled siRNA (siControl) was delivered through the RWN of mice 72 h before noise exposure. The results showed that siFasL significantly reduced the expression level of FasL in the cochlear tissue (Figure 9A). After noise exposure, FasL silencing significantly reduced the expression of FasL in OHCs by about 50% compared to siControl (Figures 9B,C), without affecting the level of NFAT3 in the nucleus (Figure 9D). Western blot results showed that FasL silencing reduced the expression levels of cleavage caspase3 and P-MLKL in both normal and noise-exposed cochlea (Figures 9E–H), suggesting that siFasL inhibited the activation of apoptotic and necroptotic signaling pathways in the cochlea. Quantification analysis of the nuclei of OHCs in the basal turn of the cochlea showed that FasL silencing significantly improved noise-induced OHCs apoptotic and necrotic nuclear changes (Figure 9I). In addition, siFasL treatment significantly attenuated the 16 kHz and 32 kHz auditory threshold shifts 2 weeks after noise damage (Figure 9J).

## 4 Discussion

**Figure 10:**
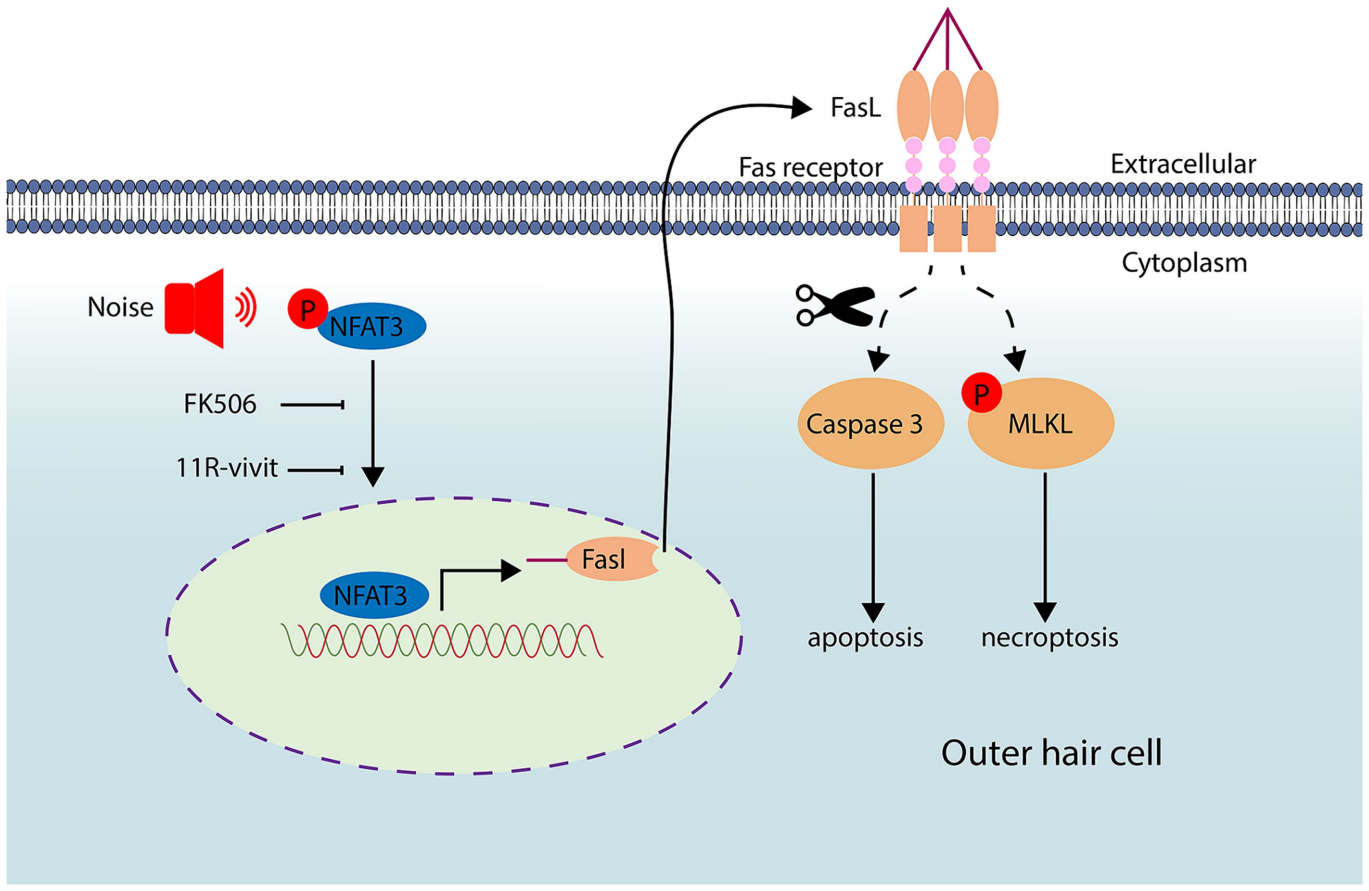
Noise exposure triggers the activation and nuclear translocation of NFAT3. Activated NFAT3 enhances the expression of FasL, which can be inhibited by administration of FK506 and 11R-vivit. Through an autocrine mechanism, FasL binds to the Fas receptor, leading to apoptosis and necroptosis of cochlear OHCs.

The precise mechanism underlying cochlear hair cell death after noise trauma remains elusive. In this study, we made important observations regarding the involvement of both apoptosis and necroptosis in the death of OHCs after noise exposure. Remarkably, this process was found to be mediated through the NFAT3/FasL signaling pathway. FasL typically activates the programmed cell death pathway in neighboring cells expressing Fas receptors. Interestingly, our findings indicate that OHCs activate their own extrinsic cell death pathway through autocrine FasL. Significantly, we demonstrated that by blocking the NFAT3/FasL signaling axis, we were able to alleviate NIHL, highlighting the potential of this pathway as a novel target for the prevention and treatment of NIHL (Figure 10).

In the cochlea, immunofluorescence staining of nuclear morphology is typically used to easily quantify apoptosis and necrosis in OHCs (Jiang et al., 2006). It has been reported that not all apoptotic cochlear hair cells were TUNEL-positive, a phenomenon that has been observed in intestinal mucosal epithelial cells as well (Jiang et al., 2006; Vyas et al., 2007). In our study, we combined nuclear morphology and cleavage caspase-3 immunofluorescence staining to collectively identify apoptotic cells. The results showed that cleavage caspase-3 was highly expressed in OHCs after noise exposure, and there was a specific enhancement around the condensed nuclei. The protein MLKL serves as the executor of necroptosis, and phosphorylated MLKL can translocate to the plasma membrane and form pores, thereby inducing cell death. The upregulation of P-MLKL expression has been documented in neuronal necroptosis associated with Parkinson’s disease (Lin et al., 2020). In our study, we observed an upregulation of P-MLKL expression in OHCs following noise trauma. Interestingly, there was a specific enhancement of P-MLKL labeling in necrotic OHCs and regions where OHCs disintegrated, suggesting the involvement of P-MLKL-mediated necroptosis in the pathogenesis of NIHL.

He et al. (2021) utilized an antibody specific for dephosphorylated NFAT3 and reported that exposure to noise resulted in the nuclear accumulation of NFAT3 in OHCs derived from CBA/J mice. Interestingly, they observed that the NFAT3 expression was also present in the nuclei of normal OHCs from control mice. In contrast, our study showed that normal OHCs only exhibited NFAT3 labeling in the cytoplasm. This discrepancy could be due to variations in the mouse strains used or differences in the antibodies employed. A recent study by Zhang demonstrated that cochlear hair cells in NFAT3-deficient mice exhibited reduced sensitivity to ototoxic drugs (Zhang et al., 2019). Consistent with previous studies but advancing further, our research demonstrated that the transcriptional activity of NFAT3 contributed to the apoptosis and necroptosis of OHCs, and that pharmacological inhibition of NFAT3 could protect against noise-induced death of OHCs.

In addition to the immune system, the FasL/Fas interaction has also been implicated in angiotensin-induced renal tubular cell death and cardiomyocyte cell death (Eid et al., 2019; Zhu et al., 2020). Bodmer et al. (2002) reported that normal murine cochlea did not express FasL, but it could be induced during sterile labyrinthitis. In another study investigating the role of Fas receptor in gentamicin ototoxicity of the organ of Corti explant, it was found that there was no significant difference in hair cell loss between Fas-deficient mice and their wild-type counterparts (Bodmer et al., 2003). Our results showed that the activation of FasL plays a crucial role in the apoptosis and necroptosis of OHCs triggered by noise exposure. Similar to our study, Bae et al. (2008) found that the normal cochlea of guinea pigs exhibited Fas expression, while FasL expression was very low. Two days after gentamicin administration, FasL expression significantly increased in the cochlea, while Fas levels remained unchanged. Furthermore, FasL was specifically expressed in the apoptotic spiral ganglion cells. Therefore, they concluded that gentamicin induces apoptosis of spiral ganglion cells via the FasL–Fas pathway.

For a long time, the involvement of the extrinsic death pathway in cochlear hair cells has remained unconfirmed. This could be attributed, in part, to the fact that the activation of the canonical extrinsic pathway typically requires the signaling of death ligands from adjacent cells. However, cochlear hair cells are surrounded by perilymph and lack direct contact with each other. A recent study has shed light on this matter by demonstrating that OHCs underwent necroptosis in response to TRAIL signaling from supporting cells in the cochlea infected by a virus (Hayashi et al., 2021). Similar to TRAIL, FasL primarily induces apoptosis in neighboring cells by binding to its receptor, Fas. Linkermann et al. (2011) conducted a study revealing that renal tubular cells underwent FasL-mediated fratricide in the context of cisplatin nephrotoxicity. However, in our study, FasL was predominantly found to be expressed in the apoptotic and necrotic OHCs themselves, rather than in adjacent OHCs or in supporting cells. In addition, according to the results of our cochlear sections and surface preparations, compared with the control group, FasL expression was only upregulated in the OHCs of the surface preparation, while there was no significant change in the expression of FasL in the remaining cochlear cells. Consequently, OHCs experienced autonomous cell death through FasL signaling after noise trauma.

Collectively, our results demonstrated that the FasL-mediated extrinsic death pathway exists in cochlear hair cells after noise trauma, the mechanism of which is contributed by the NFAT3 transcription activity and leads to downstream apoptosis and necroptosis. Our study provides new insight into the mechanisms underlying cochlear hair cell death and noise-induced hearing loss.

## Data availability statement

The original contributions presented in the study are included in the article/Supplementary material, further inquiries can be directed to the corresponding author.

## Ethics statement

The animal study was approved by Institutional Animal Care and Use Committee (IACUC) at Peking University People's Hospital. The study was conducted in accordance with the local legislation and institutional requirements.

## Author contributions

WW: Formal analysis, Funding acquisition, Methodology, Project administration, Visualization, Writing – original draft, Writing – review & editing. LY: Conceptualization, Funding acquisition, Supervision, Writing – review & editing. SL: Investigation, Methodology, Writing – original draft. LH: Funding acquisition, Investigation, Software, Writing – original draft. HZ: Funding acquisition, Project administration, Resources, Supervision, Visualization, Writing – review & editing.
