# Supplementary material for: NFAT3-FasL axis synchronously regulates apoptosis and necroptosis in murine cochlear outer hair cells after noise trauma
Source: Front Mol Neurosci. 2024 Jul 15;17:1422646. doi: 10.3389/fnmol.2024.1422646 (PMC11284637; doi:10.3389/fnmol.2024.1422646)
Supplement: Supplementary file 1 [file Table1.DOCX]

Supplementary Material

#
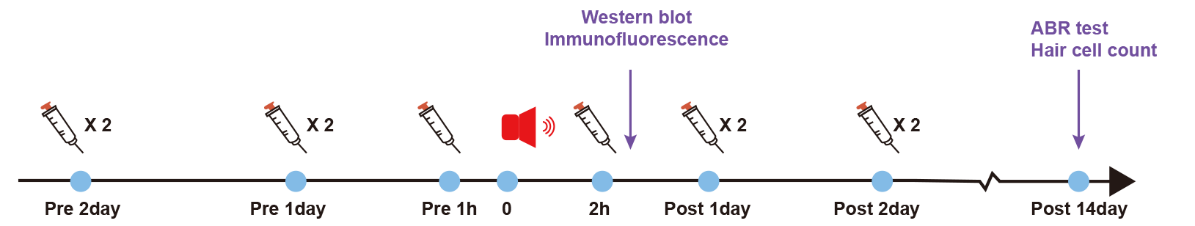
Supplementary Figures

**Figure S1.** Noise exposure and drug administration protocol. The syringe represents intraperitoneal injections of FK506 or 11R-vivit, and the speaker symbolizes a 2-hour exposure to white noise. Mice were euthanized and collected for immunofluorescence staining and western blot experiments 1-3 hours after the noise exposure according to experimental settings. ABR tests and hair cell quantification were performed 2 weeks after the noise exposure.


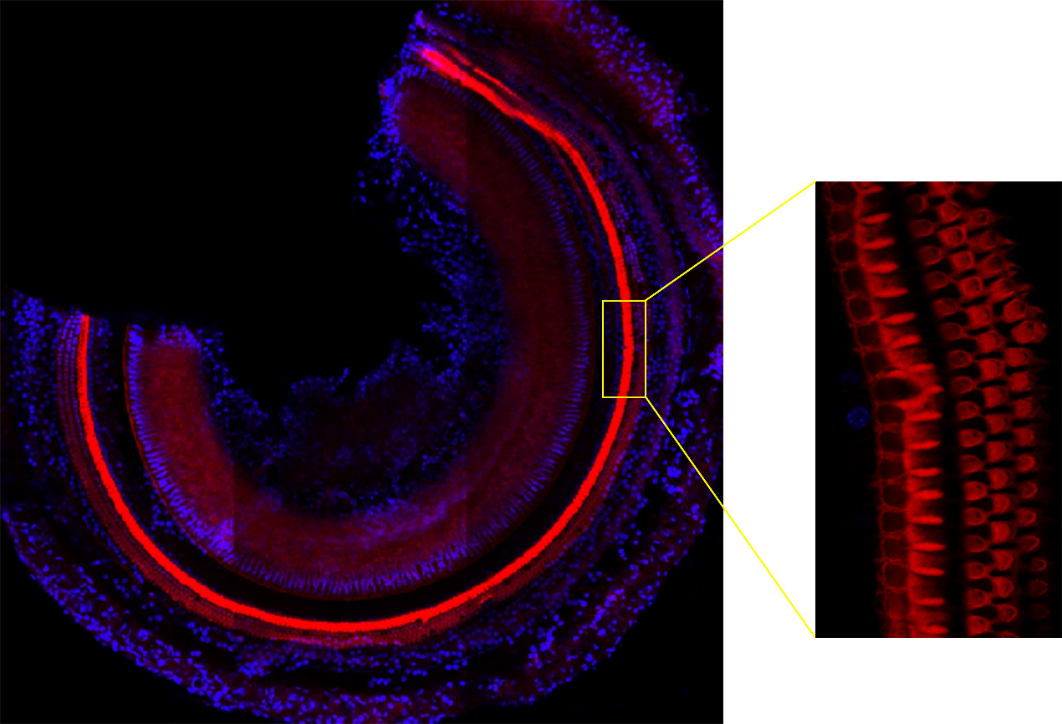
**Figure S2.** Schematic diagram of hair cell quantification for surface preparations. The image shows the middle turn of the cochlear epithelium, which was labelled with phalloidin and Hoechst33342.


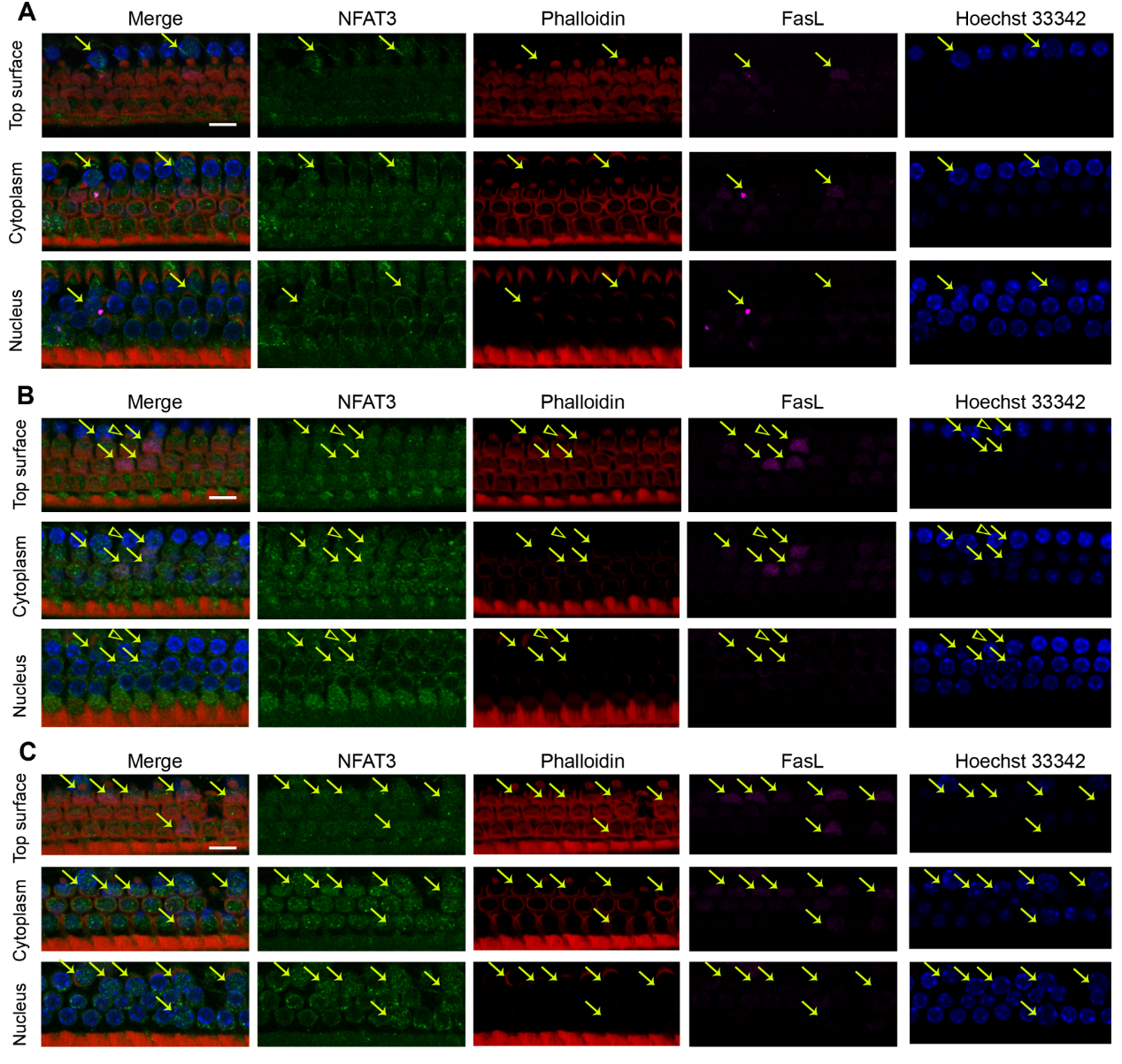


**Figure S3.** Noise exposure induces nuclear translocation of NFAT3 and high expression of FasL in apoptotic and necroptotic OHCs. Immunofluorescence staining was conducted in the basal turn of cochlear epithelia 2 h after noise exposure for NFAT3, FasL, Phalloidin, and Hoechst 33342. Panels A, B, C depict representative images from different sites with evident death of OHCs. Arrows indicate necroptotic OHCs and triangle indicate apoptotic OHCs. Scale bar = 10 μm.
